# SE-DBIRNet: Squeeze-and-Excitation Driven Dual-Path Residual Network for Mango Shelf-Life Stages Classification

**DOI:** 10.3390/foods15132279

**Published:** 2026-06-25

**Authors:** Ibrar Ahmad, Bushra Siddique, Muhammad Junaid, Mostafa Gouda, Aftab Khaliq, Zia Ul Haq, Zhengjun Qiu

**Affiliations:** 1 College of Biosystems Engineering and Food Science, Zhejiang University, Hangzhou 310058, China; ibrarrai@zju.edu.cn (I.A.);; 2 Institute of Biotechnology, Zhejiang University, Hangzhou 310058, China; 3 Department of Nutrition & Food Science, National Research Center, Dokki, Giza 12622, Egypt; 4 Agricultural Engineering Institute (AEI), National Agricultural Research Center (NARC), Pakistan Agricultural Research Council, Islamabad 44000, Pakistan; 5 Department of Farm Machinery and Precision Engineering, Faculty of Agricultural Engineering and Technology Pir Mehr, Ali Shah Arid Agriculture University, Rawalpindi 46000, Pakistan

**Keywords:** artificial intelligence, shelf-life assessment, deep learning, postharvest operations, food quality

## Abstract

Post-harvest losses of mango (*Mangifera indica* L.) in developing economies are estimated at 5% to 30%, largely due to manual management practices that depend on subjective visual assessments. This paper proposes a lightweight deep learning architecture, termed SE-DBIRNet, for real-time classification of mangoes into five shelf-life stages: unripe, semi-ripe, fully ripe, overripe, and perished. The model incorporates three key design strategies: (i) depthwise separable convolutions, achieving an 88.5% reduction in parameters when integrated into the ResNet50 backbone; (ii) a double-branch inverted residual (DBIR) module designed to enhance feature diversity and richness; and (iii) a squeeze-and-excitation (SE) attention mechanism for adaptive channel-wise recalibration. Using a public benchmark dataset of 4428 RGB images (Mendeley Data) under 10-fold cross-validation, SE-DBIRNet achieved 98.24% accuracy. Among lightweight CNN architectures (EfficientNetB0, MobileNetV2, ResNet50), SE-DBIRNet outperformed the best lightweight baseline (EfficientNetB0: 96.57%) by 1.67 percentage points. While dedicated attention-based DenseNet variants (e.g., DSA-DenseNet: 99.20%) achieved higher accuracy, SE-DBIRNet offers a superior trade-off among accuracy, inference speed (56.9 ± 1.8 FPS), and memory efficiency (8871 ± 45 MB CPU memory). EigenCAM activation visualizations revealed that the model focuses on biologically relevant and stage-discriminative features, including surface color gradients, texture uniformity, lenticel patterns, and decay boundaries. Overall, SE-DBIRNet achieves a Pareto-optimal balance among accuracy, speed, and memory efficiency, making it a strong candidate for real-time, edge-deployable post-harvest mango quality-monitoring systems, particularly when computational resources are limited.

## 1. Introduction

Food security is one of the most critical structural challenges in developing economies, and its solution demands both increased food production and improved conservation of existing food supplies [1]. Mango (*Mangifera indica* L.) is one of the most commercially and nutritionally important tropical fruits, and it is grown primarily by smallholder producers in South and Southeast Asia, Sub-Saharan Africa, and Latin America [2]. Postharvest losses of mango reach approximately 30–35%, posing a major threat to food security and farmer incomes in developing economies, primarily due to inadequate postharvest management by unskilled labor [3,4]. Real-time, accurate assessment of mango shelf life can minimize waste, enhance supply chain decisions, and boost financial returns for smallholder farmers and processors [5]. In addition, real-time recognition of mango shelf-life stages provides a strong foundation for stage-wise classification, thereby enhancing post-harvest management [6]. Each stage lends itself to different handling and utilization strategies. Unripe mangoes are suitable for pickles, salads, and international markets due to their longer shelf life during transport [7]. Semi-ripe mangoes are suitable for short-term distribution in the local market [8]. Fully ripe mangoes are best consumed fresh, command the highest market value, and are ideally suited for local markets [9]. Overripe mangoes should be used promptly, separated from sound fruit, and processed into purees, juices, or pulps. Finally, perished mangoes must be removed immediately to prevent contamination from spreading to other fruit.

A major contributor to post-harvest losses in the mango supply chain is the difficulty of accurately assessing fruit shelf-life and marketable quality [10]. The predominant approach to shelf-life assessment in developing countries, particularly Pakistan, remains visual inspection by human operators. This method is subjective, produces variable results, and is not scalable across packinghouses and distribution networks [11,12,13]. Consequently, consignments are often harvested, stored, and transported under suboptimal conditions, leading to premature spoilage, wholesale market rejections, and increased losses at the retail and consumer levels [14]. Hence, the urgent need for objective, reliable, and real-time methods to measure mango quality and determine its shelf-life stages in a scalable manner.

Although automated solutions such as multispectral sensors and traditional computer vision pipelines have shown promise, many remain expensive, demand-controlled imaging environments, or are computationally intensive, rendering them unsuitable for edge deployment [4,15]. The initial computational methods laid the groundwork for automated ripeness classification by implementing a three-step process. First, images are acquired under controlled or semi-controlled conditions. Second, the images are processed to extract handcrafted features that encode color, texture, and shape. Third, classical machine learning algorithms are applied for classification [16]. In this direction, given the low computational complexity of histogram analysis, the shelf-life stages of mango were categorized using visual features of size and color, achieving 94.97% accuracy [15]. Similarly, low-level biochemical, physical, and electrical features were integrated into a feed-forward artificial neural network (FANN), resulting in a classification accuracy of 89.6% [17]. Furthermore, 24 low-level image features were extracted from more than 100 RGB images, and a decision tree classifier was used for ripening-stage classification, achieving 96% accuracy [18].

The rise of deep learning, particularly Convolutional Neural Networks (CNNs), has revolutionized fruit quality assessment by enabling models to learn features directly from raw image data without manual feature engineering [19,20]. Deep transfer learning models, namely, VGGNet, ResNet, DenseNet, InceptionV3, MobileNetV2, and EfficientNet, have been applied to a wide range of fruit grading [21], disease detection [22], and ripeness classification tasks [23], consistently yielding higher accuracy than classical methods. Initially, to classify the ripening stages, a custom Convolutional Neural Network (CNN) was developed and trained on over 6000 RGB images, achieving a test accuracy of 76% [24]. In pursuit of higher accuracy, an InceptionNet-based model achieved 98.75% accuracy in classifying mangoes into three shelf-life stages [25]. Research has demonstrated that the external quality of fruit (skin color, firmness, and surface defects) is not always a good indicator of the ripeness stage of mangoes across different varieties [26,27].

Traditional non-destructive methods, such as total soluble solids (TSS), dry matter (DM), titratable acidity (TA), and firmness, provide accurate assessments of mango maturity but are too time-consuming and labor-intensive for high-throughput or real-time measurements [28]. In contrast, hyperspectral imaging (HSI) provides a combined spatial–spectral view that captures both external and internal fruit structures, offering a comprehensive assessment of fruit homogeneity and ripeness distribution [29,30]. Therefore, a comparison of the non-destructive methods of visible and near-infrared spectroscopy (visNIRS) and laser Doppler vibrometry (LDV) for predicting mango ripening stages was conducted. VisNIRS performed best, achieving a computed R^2^ value of 0.729 [31]. To improve the classification accuracy of mango ripening stages, color and UV-induced fluorescence images were integrated and analyzed using Partial Least Squares Regression, yielding an R^2^ of 0.97 [32]. Considerable progress has been made in non-destructive evaluation of mango ripeness; however, notable gaps persist. The majority of studies adopt unimodal approaches, including near-infrared spectroscopy (NIRS) [33], hyperspectral imaging (HSI) [34], and conventional image processing [35], whereas few investigations integrate both HSI and NIRS to simultaneously capture internal and external fruit properties.

Despite these advances, few deep learning solutions are explicitly developed for post-harvest field applications with extreme computational and latency requirements, especially in developing countries. However, very few studies have attempted to simultaneously optimize high classification accuracy, low inference latency, and minimal memory requirements for practical mango shelf-life stage recognition under realistic imaging conditions. Furthermore, the decision-making mechanisms of many models in use are not well understood, and none of these models have been studied to determine whether the learned representations are biologically meaningful and stage-discriminative visual features. This disparity hinders trustworthiness and implementation of these systems among stakeholders in the supply chain.

Furthermore, lightweight CNN models, which are predominantly used for RGB-image-based classification of mango fruits, generally rely on single-path or sequentially stacked inverted residual blocks. The proposed model introduces the double-branch inverted residual (DBIR) module, which processes features in parallel using two different kernel sizes within the same block, simultaneously capturing fine-grained textural cues and broader spatial characteristics. Similarly, integrating the squeeze-and-excitation (SE) attention mechanism with the double-branch inverted residual (DBIR) module is a novel approach. The SE block adaptively recalibrates channel-wise feature maps using combined multi-scale context, thereby enabling the network to emphasize biologically meaningful attributes. Finally, using depthwise separable convolutions in each branch, along with the DBIR architecture, reduces the number of parameters and leads to faster inference in real-world settings.

To address the research gaps we identified, we present SE-DBIRNet, a lightweight network built for real-time mango shelf-life stage recognition in real-world conditions. This study makes four main contributions. First, we introduce a new architecture that combines depthwise separable convolutions, a double-branch inverted residual (DBIR) module, and squeeze-and-excitation (SE) self-attention. This design allows for accurate multi-stage classification while keeping computational demands low. Second, we show that our approach achieves a strong balance among accuracy, inference speed, and memory usage, as demonstrated by tests on a public mango dataset of 4428 images and 10-fold cross-validation. Third, we use EigenCAM to analyze how the model focuses on biologically meaningful fruit features, such as changes in skin color and lenticel patterns, which helps build trust among users and stakeholders. Finally, we show that SE-DBIRNet is practical for real-time use in post-harvest quality monitoring systems, helping to solve a key challenge in supply chains in developing economies.

## 2. Materials and Methods

### 2.1. Data Description

**Figure 1:**
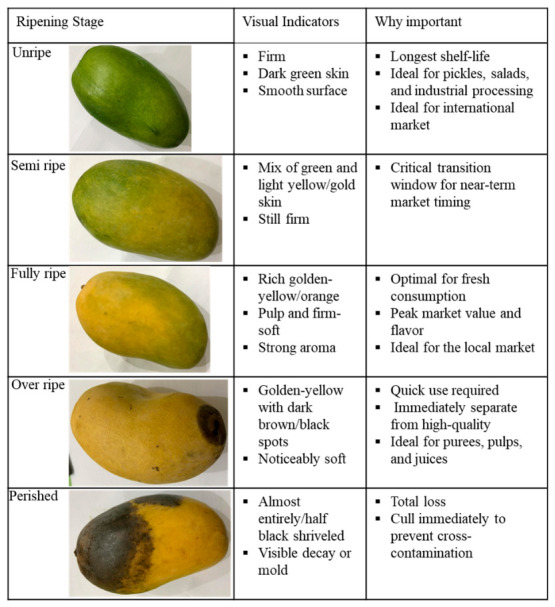
Sample images of the five distinct mango shelf-life stages.

In this study, we used a publicly available dataset acquired from the Mendeley Data Center which comprised five shelf-life stages of White Chaunsa Late mangoes [36]. The available dataset consists of 4428 color (RGB) images representing five shelf-life stages of harvested mangoes: unripe (1350), semi-ripe (1157), fully ripe (841), overripe (896), and perished (184). Figure 1 shows sample images of five shelf-life stages of mango, along with key visual indicators that help differentiate them. Table 1 presents the dataset distribution for mango ripening-stage classification.

### 2.2. Development of a Classification Model for Shelf-Life Stages of Mango

For real-time recognition of different mango shelf-life stages, this study introduced a lightweight deep learning model with higher FPS. The model, named SE-DBIRNet, is designed based on ResNet50. Initially, to make the ResNet50 lighter, the standard convolution was replaced by depth-wise separable convolution. In the next stage, a double-branch inverted residual (DBIR) module was designed by splicing two inverted residual modules in parallel. Finally, the SE attention mechanism was applied to the DBIR module to enhance the channel and spatial connectivity of features.

#### 2.2.1. Improvements in ResNet50 and Double-Branch Inverted Residue Module

The primary architecture of ResNet50 relies on numerous standard convolutional layers, which results in a substantial parameter count. In contrast, to reduce the computational burden while preserving the representational capacity of the standard ResNet50, DBIRNet employs a more efficient design using depthwise and pointwise convolutions. The mathematical formulation of this substitution is presented below. A standard convolution layer can be defined in the form of a kernel tensor as $\kappa \in {\mathbb{R}}^{k\times k\times C_{in}\times C_{out}}$, an input feature map $X\in R^{H\times W\times C_{in}}$, and an output feature map $Y\in R^{H\times W\times C_{out}}$. The standard convolution operation at the spatial location (h,w) for the output channel $c_{out}$ is expressed in Equation (1).

(1) $$Y_{h,w,c_{out}}=\sum_{i=0}^{k-1}\sum_{j=0}^{k-1}\sum_{c_{in}=1}^{C_{in}}K_{i,j,c_{in},c_{out}}\times X_{h+i,w+j,c_{in}}+b_{c_{out}}$$

where $b_{c_{out}}$ denotes the bias term associated with the $c_{out}$-th output channel. Equation (2) presents the total trainable parameters of the standard convolution layer.

(2) $$P_{stdc}=k^{2}\times C_{in}\times C_{out}$$

where $P_{stdc}$ represents the total number of parameters in the standard convolution, k is kernel size, $C_{in}$ represents the number of input channels, $C_{out}$ represents the number of output channels.

A depthwise separable convolution layer factorizes standard convolution operations into two distinct sequential steps. The first step applies depthwise convolution, followed by pointwise convolution in the second step. In a depthwise convolution, a single convolutional filter is applied independently to each input channel. A depthwise separable convolution layer can be defined by a depthwise kernel $\kappa^{\left(dw\right)}\in R^{k\times k\times C_{in}}$ and the computation expression of the depthwise output $\overset{\wedge }{Y}\in R^{H\times W\times C_{in}}$ is expressed in Equation (3).

(3) $${\overset{\wedge }{Y}}_{h,w,c}=\sum_{i=0}^{k-1}\sum_{j=0}^{k-1}K_{c}^{\left(dw\right)}\times X_{h+i,w+j,c}+b_{c}^{\left(dw\right)}$$

where $b_{c}^{\left(dw\right)}$ represents the bias for the channel c and the parameter count for this operation is expressed in Equation (4).

(4) $$P_{dw}=k^{2}\times C_{in}$$

In pointwise convolution, a 1 × 1 convolution is applied to linearly combine the depthwise outputs across channels. For a pointwise kernel $\kappa^{\left(pw\right)}\in R^{C_{in}\times C_{out}}$, and the final output is expressed in Equation (5).

(5) $$Y_{h,w,c_{out}}=\sum_{c=0}^{C_{in}}K_{c,c_{out}}^{\left(pw\right)}\times {\overset{\wedge }{Y}}_{h,w,c}+b_{c_{out}}^{\left(pw\right)}$$

where $b_{c_{out}}^{\left(pw\right)}$ is the bias for the $c_{out}$-th output channel and the trainable parameters count for pointwise convolution is expressed in Equation (6).

(6) $$P_{pw}=C_{in}\times C_{out}$$

The total number of parameters for depthwise separable convolution can be computed by summing Equations (4) and (6).

(7) $$P_{sep}=P_{dw}+P_{pw}=k^{2}\times C_{in}+C_{in}\times C_{out}$$

The parameter reduction ratio R relative to standard convolution is expressed in Equation (8).

(8) $$R=\frac{P_{sep}}{P_{stdc}}=\frac{k^{2}\times C_{in}+C_{in}\times C_{out}}{k^{2}\times C_{in}\times C_{out}}=\frac{1}{C_{out}}+\frac{1}{k^{2}}$$

For considering k=3 and typical output channels $C_{out}=256$ in the initial stages, Equation (9) expressed the numerical instantiation of the parameter reduction ratio R.

(9) $$R=\frac{1}{256}+\frac{1}{9}\approx 0.0039+0.1111=0.115$$

**Figure 2:**
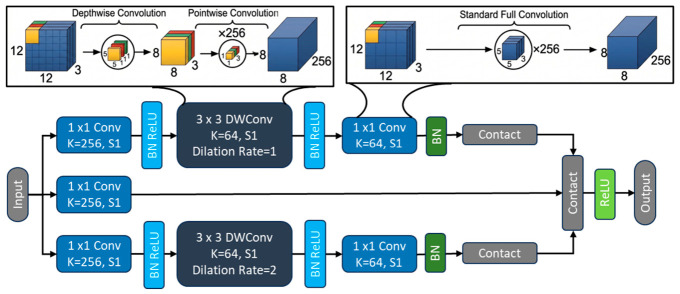
Detailed structure of the proposed multi-scale depthwise separable convolution block used within the DBIR model.

As a result, the depthwise separable convolution can reduce the number of trainable parameters by 88.5% compared to the standard convolution baseline, without reducing spatial representational capabilities. The compression is important because it enables deployment of SE-DBIRNet on mobile devices, which may be more resource-constrained than desktop devices, without sacrificing the depth needed to differentiate the various stages of mango shelf life. Figure 2 presents the schematic architecture of the DBIR model.

#### 2.2.2. SE Attention Mechanism Module

To improve attention to relevant features, the SE attention mechanism is introduced to determine the channel-wise weight relationships. It includes three steps: (1) global average pooling is used to encode the global context of the feature map, (2) a stack of fully connected layers with ReLU and Sigmoid activations is used to produce the channel weights, and (3) element-wise multiplication between the original features and the weights is used to perform channel-wise feature calibration [37]. The whole process is lightweight and easy to train, incurs no high computational cost, and consistently achieves higher accuracy in image classification. Consider an input feature map U produced by a convolutional layer, which can be mathematically defined as expressed in Equation (10).

(10) $$U\in R^{H\times W\times C}$$

where *H*, *W*, and *C* represent the height, width, and number of channels, respectively. The SE mechanism produces a recalibrated output $\tilde{U}$. The squeeze operation is a global average pooling operation that aggregates spatial information across the entire feature map for each channel, producing a scalar statistic per channel. This produces a channel descriptor $z\in R^{C}$, and the squeeze output $z_{c}$, for each channel *c*, is computed as shown in Equation (11).

(11) $$z_{c}=\frac{1}{H\times W}\sum_{i=1}^{H}\sum_{j=1}^{W}U_{c}(i,j),\;\mathrm{for}\;c=1,...,C$$

where $z_{c}$ represents the squeeze representation for the *c*-th channel, and $U_{c}(i,j)$ denotes the pixel value at the spatial location $\left(i,j\right)$ in channel *c*. A two-layer fully connected network with a bottleneck structure is used to learn channel-wise dependencies in the excitation operation, as shown in Equation (12).

(12) $$s=\sigma (W_{2}\times \delta (W_{1}\times z))$$

where $W_{1}\in R^{\frac{C}{r}\times C}$ and $W_{2}\in R^{C\times \frac{C}{r}}$ are the learnable weight matrices of the two FC layers, δ(⋅) is the ReLU activation function, σ(⋅) is the sigmoid function, r is the reduction ratio, $s\in R^{C}$ is the vector of channel-wise attention weights. The final recalibrated feature map is the rescaled original input with the learned attention weights, as shown in Equation (13).

(13) $${\tilde{U}}_{c}(i,j)=s_{c}\times U_{c}(i,j),\;\forall i,j,c$$

**Figure 3:**
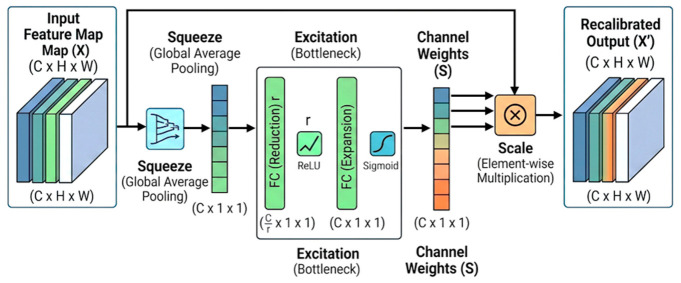
Schematic architecture of the Squeeze-and-Excitation (SE) module.

Equivalently, in tensor form:

(14) U=s⊙X

where ⊙ denotes channel-wise multiplication, and Figure 3 represents the schematic architecture of the SE module.

#### 2.2.3. Overall Architecture of the SE-DBIRNet Network

**Figure 4:**
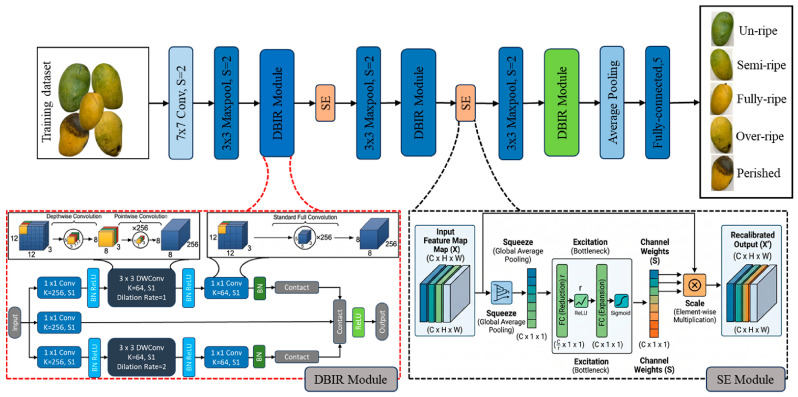
Overall architecture of the proposed SE-DBIRNet network model.

The overall architecture of the SE-DBIRNet is shown in Figure 4. The overall architecture of the SE-DBIRNet network is illustrated in the following figure. The SE-DBIRNet architecture is tailored for classifying the five shelf-life stages of mangoes, focusing on subtle changes in visual appearance, ripening progress, surface features, and overall fruit quality indicators. The first layer in the network is a 7 × 7 conv layer with a stride of 2, which maps the input mango images from 224 × 224 × 3 to 112 × 112 × 64, the beginning of feature extraction. A larger receptive field can detect initial textural patterns, color gradients, and surface irregularities, which are critical for assessing mango quality. Thereafter, a 3 × 3 max-pooling operation is performed, and 56 × 56 × 64 most discriminative features related to skin texture, color distribution, and morphology are extracted to distinguish among the fresh, ripe, overripe, and spoiled stages of mango. The main architecture consists of three hierarchical DBIR modules that run at the spatial resolutions of 56 × 56, 28 × 28, and 14 × 14. This multi-scale approach enables the network to learn surface texture details and small imperfections at high resolution, and general shape deformations and color patterns at low resolution.

The full specification of the SE-DBIRNet algorithm architecture is given in Table 2, which shows the dimensions of the input/output tensors, the kernels used by each network component, and the stride parameters. In each DBIR, spatial-specific attention maps (56 × 56 × 1 and 28 × 28 × 1) are produced with the help of squeeze-and-excitation attention mechanisms. These attention mechanisms enable the network to highlight the channels that carry the most relevant information for discriminating between the different mango shelf-life stages, especially those sensitive to color intensity, skin wrinkling, and browning patterns. The network gradually learns to ignore local textural features and focus on high-level quality features through systematic down-sampling (3 × 3 max-pooling) across the image with a stride of 2. SE modules, on the other hand, are dedicated to the most discriminative channel responses at each aspect of the feature hierarchy. The final step in classification is to transform the 14 × 14 × 64 feature representation into a small 64-dimensional descriptor that represents important quality features. The depthwise separable convolution backbone also keeps computational complexity low, while the fully connected layer maps this feature vector to five categories of mango shelf life, which is essential in the mango supply chain and for consumer safety applications such as assessing temporal quality.

#### 2.2.4. Overall Methodology for Mango Shelf-Life Stage Classification

**Figure 5:**
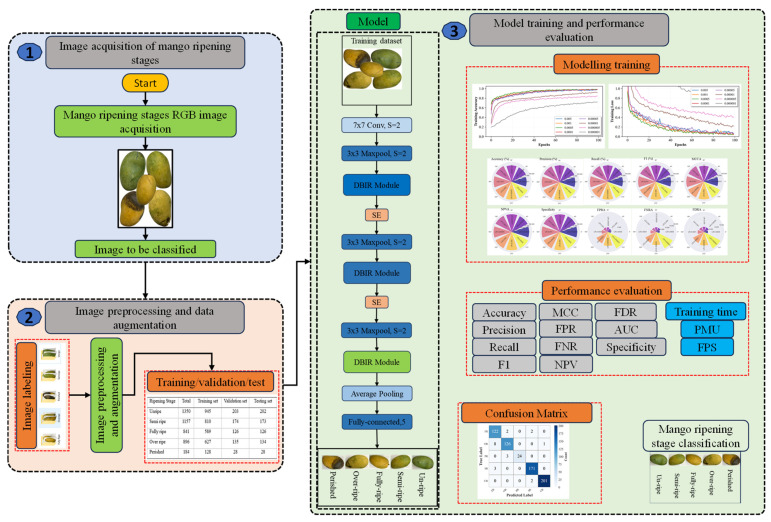
Overall methodology of mango shelf-life classification.

The overall methodology for mango shelf-life stage classification begins by obtaining RGB images of 5 ripening stages: Unripe, Semi-ripe, Fully ripe, Overripe, and Perished. These are then preprocessed and diversified for additional data. The data is then split into training, validation, and testing sets. The images are then passed through a series of convolutional and pooling layers, followed by hierarchical modules that incorporate squeeze-and-excitation attention, global average pooling, and a fully connected output layer with the five shelf-life stages. Last, training and performance testing are done with a wide variety of metrics such as accuracy, precision, recall, F1 score, specificity, PMU, FPS, FNR, FPR, NPV, AUC, MCC, and MRC, as well as a confusion matrix to get a detailed overview of the classification results from the various ripening stages. The overall methodology for classifying mango shelf-life stages is shown in Figure 5.

### 2.3. Cross-Validation Approach for Evaluation

**Figure 6:**
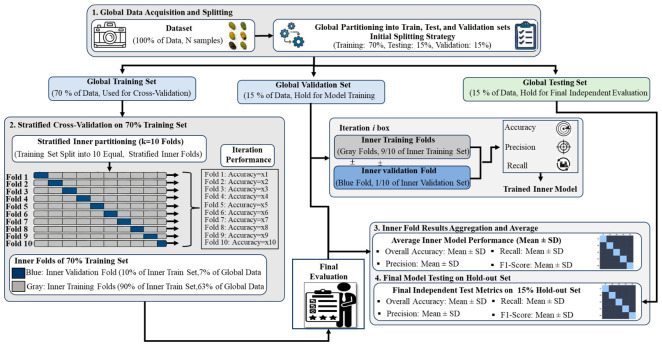
Integrated model evaluation framework with 10-fold cross-validation and independent hold-out splits.

To evaluate the performance, robustness, and generalization ability of the deep learning models for mango shelf-life recognition, a stratified 10-fold cross-validation approach was employed. In each fold, the dataset is randomly partitioned into three subsets: 70% for training, 15% for validation (used for hyperparameter tuning), and 15% for testing (used for final performance reporting). The training and validation subsets are shuffled across folds, whereas the test subset remains distinct within each fold. No additional fixed train/validation/test split is applied outside this cross-validation procedure. Table 1 presents the typical sample distribution per class within a single fold, based on the 70/15/15 split. Performance metrics are computed for each iteration, and the final results are averaged over all 10 folds. This approach mitigates bias and enhances generalizability, particularly for limited and heterogeneous datasets [38]. The process is illustrated in Figure 6.

### 2.4. Experimental Environment and Hyperparameter Configurations

#### 2.4.1. Hardware Configuration and Software Environment

The experiments were performed on a special-purpose workstation equipped with an AMD Ryzen 5 5600G processor (Advanced Micro Devices, Inc., Santa Clara, CA, USA) (3.90 GHz, 3D Radeon Graphics) and 64 GB of system RAM. The NVIDIA GeForce GTX 1060 6 GB (GDDR5) graphics card was used for GPU acceleration. The computing environment was Windows 10 Professional (64-bit), supported by CUDA-optimized deep learning libraries. To ensure efficient allocation and utilization of VRAM during simultaneous operations, GPU memory management was set to dynamic memory growth, avoiding memory allocation conflicts. To speed up computation without compromising numerical stability, especially with limited VRAM, mixed-precision training (float16/float32) was enabled using TensorFlow’s mixed-precision policy.

The deep learning pipeline is developed and trained using Python 3.12, with TensorFlow 2.19.0 as the main framework. The Keras API was the native way to build TensorFlow models, offering high-level abstractions to simplify construction, compilation, and training. The software stack included several scientific computing libraries such as NumPy (version 1.26.3) for efficient numerical array handling, Pandas (version 2.2.3) for structured data manipulation and tabulation, Matplotlib (version 3.10.3) and Seaborn (version 0.13.2) for detailed visualization of training dynamics and performance metrics, and scikit-learn (version 1.8.0) for data preprocessing, label encoding, and calculation of evaluation metrics, including precision, recall, F1-score, and ROC-AUC. Other utilities included the tqdm library (version 4.67.1), which enabled real-time progress logging for data loading and training iterations, and the GPUtil library (version 1.4.0), which allowed continuous monitoring and recording of GPU usage and temperature.

#### 2.4.2. Training Hyperparameters and Configuration

The SE-DBIRNet model was trained with a well-tuned hyperparameter set that enhances convergence stability and generalization performance. The full hyperparameter set used during training is shown in Table 3.

### 2.5. Performance Evaluation Metrics

In a deep learning framework, training and testing are considered two integral processes. A deep learning model is a machine learning model trained to learn relationships and representations of features in data. The evaluation of the model follows the training phase and is known as the test phase, where the model is evaluated on unseen data to assess its generalization and overall performance. A wide range of performance measures derived from a confusion matrix was used to evaluate the proposed SE-DBIRNet model. The confusion matrix was used to calculate class-specific metrics, including precision, recall, accuracy, Matthews Correlation Coefficient (MCC), False Discovery Rate (FDR), False Negative Rate (FNR), False Positive Rate (FPR), Negative Predictive Value (NPV), and specificity. The classification of mango shelf-life stages is formalized in Table 4. All calculations for these crucial parameters are presented in Equations (15)–(18).

**Table 4:** **Definition of confusion matrix parameters.**

Parameter	Symbol	Definition
True Positive	TP	True class is X, and the DL model predicts X
True negative	TN	True class is Y, and the DL model predicts Y
False Positive	FP	True class is Y, and the DL model predicts X
False Negative	FN	True class is X, and the DL model predicts Y

Note: In this notation, X represents the specific positive class of mango shelf-life, while Y represents any class other than X. This formulation is applicable to multi-class classification problems using a one-versus-rest approach.

(15) $$PT_{X}=c_{xx}$$

(16) $$FP_{X}=\sum_{i=1,i\neq X}^{5}c_{Xi}$$

(17) $$FN_{X}=\sum_{i=1,i\neq X}^{5}c_{iX}$$

(18) $$TN_{X}=\sum_{i=1}^{5}\sum_{j=1}^{5}c_{ij}-(TP_{X}+FP_{X}+FN_{X})$$

For the five-class classification, the confusion matrix is expressed in Equation (19).

(19) $$\begin{array}{c} C=\left[\begin{array}{ccccc} c_{11} & c_{12} & c_{13} & c_{14} & c_{15}\\ c_{21} & c_{22} & c_{23} & c_{24} & c_{25}\\ c_{31} & c_{32} & c_{33} & c_{34} & c_{35}\\ c_{41} & c_{42} & c_{43} & c_{44} & c_{45}\\ c_{51} & c_{52} & c_{53} & c_{54} & c_{55} \end{array}\right] \end{array}$$

where $c_{ij}$ represented the count of samples from the true class i predicted as class j. The diagonal elements of the confusion matrix correspond to the true positives (TP) for each class, while the off-diagonal elements represent misclassifications.

Utilizing Equations (15)–(18), the performance evaluation parameters for the specific class *X* can be computed as follows:

(20) $${\mathrm{Precision}}_{X}=\frac{TP_{X}}{TP_{X}+FP_{X}}*100$$

(21) $${\mathrm{Recall}}_{X}=\frac{TP_{X}}{TP_{X}+FN_{X}}*100$$

(22) $$Accuracy=\frac{TP_{X}+TN_{X}}{TP_{X}+FN_{X}+TN_{X}+FP_{X}}*100$$

(23) $$MCC_{X}=\frac{\left(TP_{X}*TN_{X})-(FP_{X}*FN_{X}\right)}{\sqrt{\left(TP_{X}+FP_{X})*(FP_{X}+TN_{X})*(TN_{X}+FP_{X}\right)}}*100$$

(24) $$FPR_{X}=\frac{FP_{X}}{FP_{X}+TN_{X}}*100$$

(25) $$FNR_{X}=\frac{FN_{X}}{TP_{X}+FN_{X}}*100$$

(26) $$NPV_{X}=\frac{TN_{X}}{TN_{X}+FN_{X}}*100$$

(27) $$FDR_{X}=\frac{FP_{X}}{TP_{X}+FP_{X}}*100$$

(28) $$Specificity_{X}=\frac{TN_{X}}{TN_{X}+FP_{X}}*100$$

## 3. Results and Discussions

### 3.1. Performance Comparison of the Model by Learning Rate

**Figure 7:**
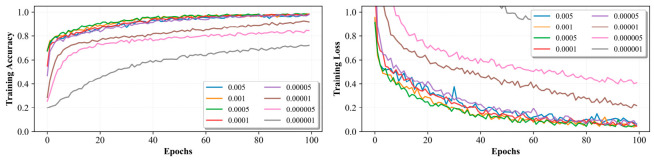
Training accuracy and training loss curves of SE-DBIRNet across eight learning rates (0.005, 0.001, 0.0005, 0.0001, 0.00005, 0.00001, 0.000005, 0.000001) over 100 epochs.

**Figure 8:**
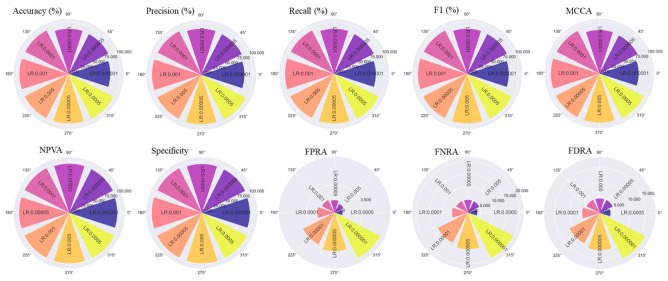
Comparative performance of SE-DBIRNet under different learning rates across multiple metrics.

The learning rate significantly impacts model performance and is a key factor in successful training. A high learning rate led to convergence issues and oscillation around the optimum, whereas a low learning rate slowed convergence and increased training time. Therefore, a comparative experiment with different learning rates was conducted on the SE-DBIRNet Architecture to determine the optimal learning rate. The initial learning rate of the proposed model was 0.005, which was gradually reduced to 0.000001, and eight learning rates were systematically considered for comparative analysis. Figure 7 shows a comparative analysis of training behavior for the proposed deep learning model at different learning rates. The training curves clearly demonstrate that the learning rate substantially affects both convergence dynamics and overall training performance. Results demonstrate that higher learning rates yielded the highest training accuracies and the fastest loss reduction. Lower learning rates led to slower convergence and lower accuracy. Based on the training curves, 0.0005 was identified as the optimal initial learning rate for the deep learning model proposed in this study. Furthermore, as shown in Figure 8, the comparative analysis of performance metrics indicates that a learning rate of 0.0005 is optimal.

### 3.2. Training Dynamics Analysis

**Figure 9:**
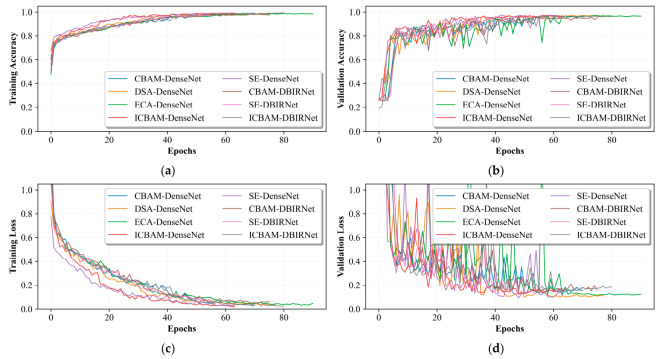
Training dynamics of hybrid deep learning models: (**a**) training accuracy; (**b**) validation accuracy; (**c**) training loss; (**d**) validation loss.

The comparative evaluation of attention-augmented architectures for real-time on-farm mango shelf-life classification is summarized in Figure 9 and Table 5. Comparative evaluation of the attention-augmented architectures for real-time on-farm mango shelf-life classification is shown in Figure 9 and Table 5. In particular, the evaluation parameters presented in Table 5 were derived from the training and validation stages of model development. Although the highest accuracy (99.29%) was achieved with ICBAM-DenseNet at the optimal learning rate of 0.0005, the proposed SE-DBIRNet offers a good trade-off between classification accuracy and computational speed, which is important for on-farm implementation. SE-DBIRNet also achieves fast convergence, achieving the best accuracy at epoch 53, compared to the high complexity ICBAM-based models (requiring 64–81 epochs). This makes SE-DBIRNet particularly well-suited for resource-constrained agricultural environments where real-time processing and low resource requirements are crucial.

The training dynamics, as shown in Figure 9a–d, demonstrate that SE-DBIRNet experiences a 5.40% reduction in convergence efficiency. However, this trade-off is justified by significant decreases in both training time and computational resource demands. These reductions are essential for future mango shelf-life assessment systems intended to run continuously in farm environments with limited processing capacity. A sensitivity analysis conducted at a learning rate of 0.0005 yielded improved performance across all evaluated architectures. Moreover, the slight 2% decrease in accuracy of SE-DBIRNet compared to ICBAM-DenseNet is offset by a remarkable 33% reduction in the number of training epochs required. This trade-off highlights the effectiveness of SE-DBIRNet in enhancing computational efficiency and reducing inference times. These parameters are essential for enabling rapid, evidence-based decisions regarding shelf-life in agricultural productivity and post-harvest management.

### 3.3. Comparative Analysis Based on Performance Metrics

Table 6 compares eight deep learning models using various attention mechanisms. It was found that there was a significant difference in classification performance between DenseNet- and DBIRNet-based architectures across multiple evaluation metrics. Among all models, DSA-DenseNet emerged as the superior architecture, achieving the highest accuracy of 99.20% ± 0.16%, followed closely by ECA-DenseNet (98.98% ± 0.16%) and SE-DenseNet (98.97% ± 0.07%). Within the DBIRNet-based architectures, ICBAM-DBIRNet achieved the highest accuracy at 98.84% ± 0.10%, while SE-DBIRNet recorded the lowest at 98.24% ± 0.09%. Precision and recall results also demonstrated the excellent performance of DSA-DenseNet, with averages of 98.23% ± 0.86% for precision and 98.23% ± 0.86% for recall. This means that the model is not overly prone to producing false positives or false negatives.

These results were further supported by the Matthews correlation coefficient (MCC), which provides a balanced assessment for imbalanced datasets. DSA-DenseNet achieved the highest MCC score at 97.43% ± 0.17%, whereas SE-DBIRNet recorded the lowest at 94.64% ± 0.11%. In the false positive rate (FPR) analysis, DSA-DenseNet had the lowest FPR (0.51% ± 0.0006%), while CBAM-DBIRNet had the highest (0.92% ± 0.0009%). Similarly, the false negative rate (FNR) analysis revealed that DSA-DenseNet had higher sensitivity (FNR of 2.11% ± 0.0023%) than the SE-DBIRNet (FNR of 4.06% ± 0.0054%). The negative predictive value (NPV) was also consistently high across all models, ranging from 98.93% to 99.45%, with DSA-DenseNet achieving the highest value of 99.45% ± 0.12%. Lastly, the AUCs for all models exceeded 0.998, with ECA-DenseNet achieving the highest (0.9993 ± 0.0008), indicating excellent discriminative ability across models.

Due to its dual attention mechanism, DSA-DenseNet achieved superior performance (99.20% ± 0.16% accuracy), along with the highest F1-score (98.21% ± 0.86%) and the lowest false discovery rate (FDR: 2.08% ± 0.0038%). The dual attention pathway aligns well with DenseNet’s dense connectivity pattern, and DSA effectively captures fine-grained spatial dependencies while focusing on discriminative regions. This interaction enables simultaneous feature reuse and spatial feature emphasis, effectively balancing the sensitivity-specificity trade-off that is crucial for robust classification systems. ECA-DenseNet was slightly outperformed by DSA-DenseNet, achieving a relatively high accuracy of 98.98%, but with the highest AUC of 0.9993 ± 0.0008 and an excellent standard deviation of 98.98% ± 0.16%. The channel-wise attention mechanism of ECA-DenseNet naturally captures inter-channel relationships with only a few extra computations and is well-suited to the concatenated-feature design of DenseNet. It does not, however, have the spatial information available in DSA’s two parallel streams of spatial attention.

Moreover, the performance gap between DenseNet- and DBIRNet-based architectures is consistently observed, further emphasizing an important architectural consideration. The accuracy of DenseNet models was typically about 0.36% higher than that of their DBIRNet counterparts. This means that DenseNet’s dense connectivity is better suited to the high-quality propagation of information between layers and the reuse of features, resulting in richer feature maps that are better processed by attention mechanisms. By contrast, the DBIRNet architecture, despite its specific imaging-task design, shows no significant gain from integrating attention into its key features, indicating a relatively weak interaction with the attention mechanism. The results show that different base architectures affect both the attention mechanism’s performance and the overall model’s performance.

### 3.4. Confusion Matrix Analysis

**Figure 10:**
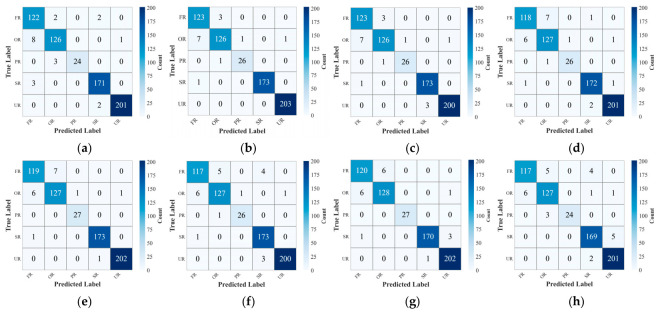
Confusion matrices for (**a**) CBAM-DenseNet, (**b**) DSA-DenseNet, (**c**) ECA-DenseNet, (**d**) ICBAM-DenseNet, (**e**) SE-DenseNet, (**f**) CBAM-DBIRNet, (**g**) ICBAM-DBIRNet, and (**h**) SE-DBIRNet.

The detailed classification performance of the attention-based hybrid models across the five mango shelf-life stages, as shown in their confusion matrices, is presented in Figure 10. The results of the confusion matrix are presented concisely using abbreviations. In particular, the five stages of mango shelf life are shortened as: Unripe (UR), Semi-ripe (SR), Fully ripe (FR), Overripe (OR), Perished (PR). The confusion matrix results showed that the DenseNet-based hybrid models performed best in diagonal dominance (the higher the better) and true positive rate (TPR) across all classes, with the DSA-DenseNet model achieving the highest performance. ECA-DenseNet had similar performance to DSA-DenseNet, with a slight decrease in the true positive rate for the UR class. CBAM-DenseNet also performed well in terms of true positive rate, especially for the UR class, but showed slight misclassification of the FR and OR classes. Furthermore, ICBAM-DenseNet and SE-DenseNet showed a slight decrease in true positive rate for FR classification accuracy compared to the DSA variant. Notably, SE-DenseNet emerged as the best performer in recognizing PR samples. Among the DBIRNet-based hybrid models, CBAM-DBIRNet showed a drop in FR accuracy (117 true positives), with six OR samples misclassified as FR and five FR samples misclassified as OR. In contrast, ICBAM-DBIRNet improved accuracy for the FR class (120 true positives) and achieved the highest true-positive count for the OR class (128). Finally, SE-DBIRNet was the least-performing model, particularly in SR (169 true positives) and FR (117 true positives), compared with the best-performing models.

Across all eight models, the UR (Unripe) class was the most consistently and accurately predicted, with true positive counts ranging from 200 to 203. The PR (Perished) class, despite having the smallest dataset support (approximately 27 samples), was classified with near-perfect accuracy by most models. The primary source of error across all architectures was confusion between the FR (Fully Ripe) and OR (Overripe) stages. Fully ripe and early overripe mangoes look very similar, which often causes confusion. Fully ripe mangoes are usually evenly yellow and slightly soft, while overripe ones show more yellow, some brown spots, and are even softer. The change between these stages happens slowly, so even experts may find it hard to tell them apart. Also, since color is a key feature of natural images, the colors of these two stages often overlap, making it hard to distinguish them from RGB images alone.

From an architectural point of view, DenseNet-based hybrid models generally exhibited better feature propagation and reuse than DBIRNet-based models, enabling them to capture subtle changes in mango color and texture across shelf-life stages. DSA-DenseNet stands out as a top performer among these; its Dynamic Sparse Attention module captures fine-grained, salient features more effectively than other modules such as CBAM and SE. Interestingly, the misclassifications across all models reflect the physical reality of the continuous biological ripening process, and are primarily limited to the neighboring Fully Ripe (FR) and Overripe (OR) stages, which are physiologically ambiguous. In contrast, the models are very accurate at the extremes of development—the Unripe (UR) stage, where properties are markedly different and highly homogeneous, and the Perished (PR) stage, where properties are markedly degraded and highly visible.

During training, class imbalance was not directly addressed. Instead, stratified cross-validation was used to keep the data’s natural distribution. According to the SE-DBIRNet confusion matrix, the accuracy for the perished class was 88.9%. This is lower than most other classes, probably because there were fewer samples in the perished category.

### 3.5. Comparative Computational Complexity Analysis of Models

**Figure 11:**
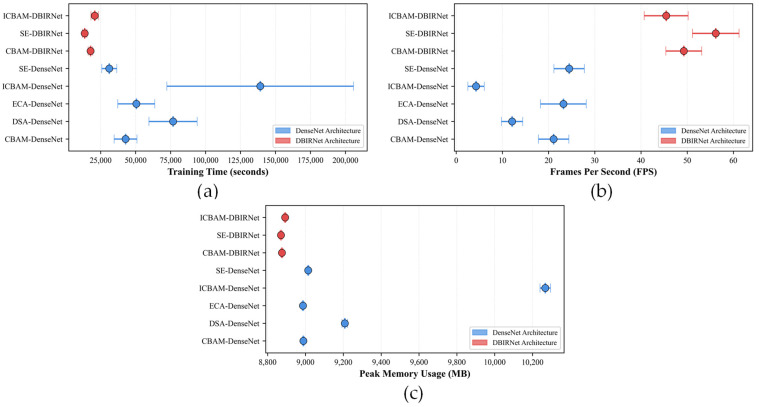
Comparative computational complexity analysis of models, (**a**) Training time (seconds), (**b**) Frames per second, (**c**) Peak memory usage.

A detailed complexity assessment of 8 architectures with enhanced attention was conducted using three metrics: total training time (seconds), frames per second (FPS), and peak memory usage (megabytes). These parameters play key roles in deploying an Artificial Intelligence-based system to classify mango shelf-life stages in real time. The lower the training time, the less the computational resources will be used, and the higher the FPS, the faster the processing speed will be. In addition, lower peak memory consumption will enable its use on mobile devices or in farm management systems. The results of the complexity analysis of the eight hybrid models are shown in Figure 11.

The SE-DBIRNet was the most efficient overall, with a convergence time of 13,311 ± 412 s, the highest inference speed of 56.9 ± 1.8 FPS, and the lowest memory consumption of 8871 ± 45 MB. CBAM-DBIRNet and ICBAM-DBIRNet required longer training times (17,953 ± 523 s and 20,164 ± 611 s, respectively) and still achieved excellent real-time performance (48.8± 1.5 FPS and 46.5 ± 1.4 FPS, respectively), while keeping memory usage at 8876 ± 52 and 8895 ± 48 MB. All DenseNet-based models, on the other hand, achieved significantly poorer trade-offs than DBIRNet. Specifically, SE-DenseNet and CBAM-DenseNet required 35,433 ± 1024 and 40,205 ± 1187 s, respectively, to converge, while delivering only 22.2 ± 0.7 and 22.7 ± 0.7 FPS, with memory usage rising to 9015 ± 63 and 8989 ± 59. The training times for DSA-DenseNet and ICBAM-DenseNet, however, were too high, at 70,432 ± 1876 s and 145,601 ± 3245 s, respectively. In addition, memory consumption was also significant: DSA-DenseNet used 9712 ± 76 MB while ICBAM-DenseNet used 10,270 ± 98 MB.

We see significant differences in 10-fold cross-validated stability between architectures in the standard deviations. The DBIRNet-based models exhibited relatively low variability, with coefficients of variation (CVs) ranging from 2.5% to 3.5% across all evaluated metrics. This means that the inferences are consistent and stable for different initializations and data partitions. The DenseNet-based models had significantly higher absolute standard deviations in training time of ±3245 and ±1876 s, respectively. This increased variability indicates greater sensitivity to random seeds and batch compositions during optimization. This instability also makes it more difficult to deploy these models, as they cannot be reliably reproduced without extensive tuning.

The mean complexity profiles show that SE-DBIRNet is the best option for real-time applications with low resource requirements and fast convergence. ICBAM-DBIRNet is a good alternative when attention mechanisms are needed more, as it requires only a small amount of training time and very little memory overhead. The DenseNet family has only two models with mean training times < 12 h: SE-DenseNet and CBAM-DenseNet. Both, however, have low mean FPS (~22) and are not suitable for near-real-time applications. By comparison, ICBAM-DenseNet and DSA-DenseNet are clearly sub-optimal, taking more than 19 h to train and yielding only less than 13 FPS and not enough of an increase in accuracy (more than 10–15 percentage points) to warrant the time. These longer average training times are a further verification of the difficulty of the training. Moreover, the low standard deviations suggest that this difficulty is also associated with poor reproducibility. SE-DBIRNet is not the most accurate model, as DSA-DenseNet achieves 99.20%. However, SE-DBIRNet offers a good balance of accuracy (98.24%), inference speed (56.9 FPS), and memory use (8.9 GB CPU memory). This balance makes SE-DBIRNet a strong choice for real-time edge deployments with limited resources, whereas DSA-DenseNet may require excessive computational power.

### 3.6. Performance Comparison of the Proposed Model with State-of-the-Art Classification Models

The results in Table 7 demonstrate that SE-DBIRNet is a robust and efficient classification model for mango shelf-life assessment, outperforming existing architectures. The model achieves the highest accuracy (98.236% ± 0.086), and the superscript letters confirm that this improvement is statistically significant compared to all other models (*p* < 0.001). For precision, recall, and F1-score, SE-DBIRNet is statistically comparable to EfficientNetB0 and ResNet50 (sharing the same letter ‘a’) but significantly better than the remaining models. The very small standard deviations across all metrics indicate excellent stability and reproducibility. The integration of the Squeeze-and-Excitation (SE) attention mechanism into the DBIRNet architecture enhances feature extraction and utilization, directly contributing to the superior classification performance. The balanced trade-off between high predictive accuracy and computational efficiency (competitive inference time and lowest memory usage) makes SE-DBIRNet highly suitable for real-world, automated mango shelf-life assessment systems.

SE-DBIRNet stands out for its efficient use of resources. It has just 2.85 million parameters, much fewer than DenseNet201 (20.3 M), ResNet50 (23.6 M), and VGG16 (134.3 M). The model needs only 0.89 GFLOPs, which is close to EfficientNetB0 (0.78 GFLOPs) and much less than InceptionV3 (5.73 GFLOPs) or Xception (8.40 GFLOPs). Its size is 10.86 MB, second only to MobileNetV2 (8.64 MB) among the models compared, but it achieves higher accuracy. Adding the Squeeze-and-Excitation (SE) attention mechanism to DBIRNet improves feature extraction and utilization, leading to better classification results. With its strong balance between accuracy and efficiency (low GFLOPs, small size, and minimal memory usage, as shown in Table 7), SE-DBIRNet is well-suited for automated mango shelf-life assessment, especially where resources are limited.

### 3.7. Interpretation of EigenCAM Activation Visualizations

**Figure 12:**
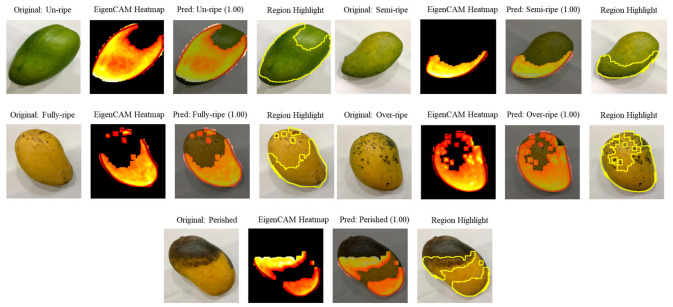
EigenCAM activation maps highlighting the regions of interest in mango images at different ripening stages.

The EigenCAM activation visualization of five harvested mangoes in different shelf-life stages (unripe, semi-ripe, fully ripe, overripe, and perished) is shown in Figure 12. The trained SE-DBIRNet effectively segments distinct regions of mango, as shown in this visualization, by accounting for surface texture and color features. The unripe mango was shown to the trained model using EigenCAM visualizations, revealing that the model predicted the uniform green surface and the smooth texture of the mango skin. At the semi-ripe stage, focus turns to yellow patches and clusters of lenticels. The proposed hybrid model highlights the richness of golden color at the fully ripe stage and the darkening at the overripe stage, with activations focusing on decay and darkening during the overripe stage. Lastly, the death stage is evident in the startling degree of decay, including changes in texture and color. The images generated by the EigenCAM results provide clear visual data on the model’s performance and show distinct visual characteristics that can assist in distinguishing mango ripening stages.

**Figure 13:**
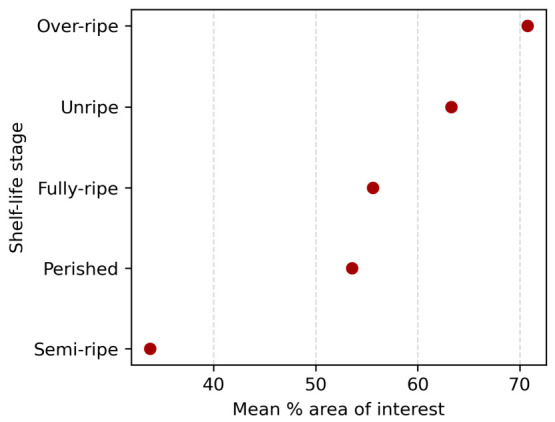
Quantitative EigenCAM activation analysis across five mango shelf-life stages.

Furthermore, to check how well SE-DBIRNet can be interpreted, we measured the average percentage of fruit surface area with EigenCAM activation above the 75th percentile for each shelf-life stage. We used 20 random images per class, for a total of 100 images and results are presented in Figure 13. The region of interest (RoI) was significantly different across the stages (one-way ANOVA, *p* < 0.001): over-ripe (70.8%), unripe (63.3%), fully ripe (55.6%), perished (53.6%), and semi-ripe (33.8%). These results match what is known about mango ripening. The small RoI for semi-ripe fruit (33.8%) shows the model focuses on the blush or shoulder area, where chlorophyll breaks down. The large RoI for overripe fruit (70.8%) is consistent with the widespread browning and softening observed at this stage. The intermediate RoI values for fully ripe (55.6%) and perished (53.6%) fruit suggest that the model focuses on evenly yellowed or orange-peeled areas and specific necrotic spots. The high RoI for unripe fruit (63.3%) matches the even green peel, which has no single hotspot. These EigenCAM results show that SE-DBIRNet learns features that make biological sense at each stage, which helps build trust in its use for edge deployment.

### 3.8. External Validation on an Independent Mango Dataset

To strengthen the generalizability of SE-DBIRNet to an independent dataset, the performance of the proposed model was assessed on an external online dataset comprising mango samples across four shelf-life stages [39]. Furthermore, to assess the robustness of the proposed deep learning model on an independent dataset, a 10-fold cross-validation approach was employed. Table 8 summarizes the comprehensive performance of SE-DBIRNet on the independent dataset, using the same evaluation parameters applied during model assessment. The results revealed that SE-DBIRNet achieved 100% accuracy in most folds, with the remaining folds also achieving accuracies exceeding 99%, demonstrating consistent performance across stages. These findings indicate that SE-DBIRNet captures robust, generalizable features that are not tied to a specific dataset.

## 4. Conclusions

The primary objective of this study was to develop a low-power, real-time deep learning network for the automated classification of the five stages of mango shelf life with high accuracy, subject to the constraints of post-harvest processing applications. The proposed SE-DBIRNet achieved 98.24% ± 0.09% classification accuracy, 1.67 percentage points higher than the 8 state-of-the-art baselines, including EfficientNetB0 (96.57%). More importantly, this enhanced accuracy was matched by the fastest inference speed (56.9 ± 1.8 FPS), lower peak memory usage (8871 ± 45 MB), and 88.5% fewer parameters than ResNet50. A near-perfect cross-fold consistency is illustrated by the very low standard deviation in accuracy (±0.09%). In addition, biologically meaningful features such as color gradients, texture uniformity, lenticel patterns, and decay boundaries were identified using EigenCAM visualizations, thereby improving the model’s interpretability for real-world use.

From the practical perspective, SE-DBIRNet is a potential solution for edge deployment in post-harvest sorting lines. It is very accurate, uses less memory, and is very fast, which can reduce manual sorting work and food waste. Furthermore, the systematic use of depthwise separable convolution, double-branch inverted residual module, and squeeze-and-excitation attention demonstrates efficiency without compromising discriminative power. The design methodology could be applicable to other fine-grained visual recognition applications in resource-constrained systems.

While SE-DBIRNet performs well, there are some practical limitations to consider. First, the dataset in this study includes only one mango variety, White Chaunsa Late, and all images were taken in a studio with consistent lighting and background. This means the model may not work as well with other mango types, such as Sindhri, Langra, or Dosehri, or in real-world farm or post-harvest settings where lighting and backgrounds are less controlled. Second, the dataset is imbalanced, with 184 images for the Perished class and 1350 for the Unripe class. This could affect how well the model performs for each class. For example, the accuracy for the Perished class was 88.9%, lower than that of most other classes. Third, even though SE-DBIRNet has a fast inference time (0.022 s per image) and uses little memory (8892 MB), deploying it on devices with limited resources, such as smartphones or Raspberry Pi, would require additional optimizations, such as quantization and pruning, to meet real-time speed and power requirements. Finally, the model uses only RGB images, which capture only surface features. It cannot measure internal qualities like sugar content (Brix), firmness, or internal browning.

To overcome these limitations, future research could take several directions. First, the database could be expanded to include more mango varieties and real-world lighting, possibly using domain adaptation to close the gap between studio and field settings. Second, strategies to address class imbalance, like class-weighted loss functions or targeted data augmentation for the Perished class, could be added. Third, SE-DBIRNet could be tested on edge devices such as the NVIDIA Jetson Nano or Raspberry Pi, using model optimization methods such as quantization, pruning, or knowledge distillation to enable real-time use in conveyor-belt sorting or handheld tools. Fourth, the approach could be extended to use multimodal data, such as near-infrared or hyperspectral imaging, to detect internal quality features that are not visible on the surface. Exploring ordinal regression may also help better capture the natural order of ripening stages (unripe to perished) and reduce confusion between similar classes. Finally, SE-DBIRNet offers a pre-optimized design that can be adapted to other tropical fruits, such as banana, papaya, and guava, and can serve as a template for building efficient and interpretable fruit-quality monitoring systems.

## Figures and Tables

**Table 1:** Data splits by ripening stage for optimal learning rate analysis, and sample distribution per ripening stage within a single fold of the 10-fold cross-validation.

Ripening Stage	Total	Training Set	Validation Set	Testing Set
Unripe	1350	945	203	202
Semi ripe	1157	810	174	173
Fully ripe	841	589	126	126
Over ripe	896	627	135	134
Perished	184	128	28	28

**Table 2:** Detailed architecture of SE-DBIRNet algorithm.

Module Name	Input Shape	Output Shape	Kernel Size	Stride
Conv	224 × 224 × 3	112 × 112 × 64	7 × 7	2
Maxpool	112 × 112 × 64	56 × 56 × 64	3 × 3	2
DBIR Module	56 × 56 × 64	56 × 56 × 64	_	_
SE Module	56 × 56 × 64	56 × 56 × 1	_	_
Maxpool	56 × 56 × 64	28 × 28 × 64	3 × 3	2
DBIR Module	28 × 28 × 64	28 × 28 × 64	_	_
SE Module	28 × 28 × 64	28 × 28 × 1	_	_
Maxpool	28 × 28 × 64	14 × 14 × 64	3 × 3	2
DBIR Module	14 × 14 × 64	14 × 14 × 64	_	_
Average pooling	14 × 14 × 64	1 × 1 × 64	_	_
Fully connected	64	5	_	_

**Table 3:** Hyperparameter Configuration for SE-DBIRNet Training.

Category	Hyperparameter	Value
Optimization	Algorithm	Adam (Adaptive Moment Estimation)
	Initial Learning Rate	5.0 × 10^−4^
	β_1_ (momentum)	0.9
	β_2_ (momentum)	0.999
	ε (epsilon)	1.0 × 10^−7^
Learning Rate Schedule	Scheduler Type	ReduceLROnPlateau
	Monitoring Metric	Validation Loss
	Reduction Factor	0.5
	Patience	7 epochs
	Minimum Learning Rate	1.0 × 10^−7^
	Batch Size	32
	Input Dimensions	224 × 224 × 3 pixels
	Maximum Epochs	100
	Loss Function	Sparse Categorical Cross-Entropy
Early Stopping	Status	Enabled
	Monitoring Metric	Validation Loss
	Patience	15 epochs
	Minimum Delta	1.0 × 10^−4^
	Restore Best Weights	Yes
Regularization	Weight Initialization	He Normal
	Dropout Rates (cascaded)	0.5 → 0.3 → 0.2
	Batch Normalization	After each convolutional layer
Data Processing	Augmentation Strategy	On-the-fly (training set only)
	Preprocessing	DenseNet-specific normalization
Model Output	Activation Function	Softmax
	Precision Type	Float32
Performance Optimization	Mixed Precision Training	Enabled (mixed_float16)
	Model Checkpointing	Best validation accuracy
	GPU Memory Growth	Dynamic allocation

**Table 5:** Training dynamics of convergence analysis.

Model Name	Final Accuracy (%)	Best Accuracy (%)	Convergence Efficiency (%)	Total Training Epochs	Epochs to Best Accuracy
CBAM-DenseNet	98.90	99.13	1.40	71.00	70.00
DSA-DenseNet	98.61	98.84	2.60	78.00	76.00
ECA-DenseNet	98.39	98.94	4.40	91.00	87.00
ICBAM-DenseNet	99.29	99.29	0.00	64.00	64.00
SE-DenseNet	99.06	99.06	0.00	64.00	64.00
CBAM-DBIRNet	97.64	98.42	7.90	76.00	70.00
SE-DBIRNet	98.30	98.45	5.40	56.00	53.00
ICBAM-DBIRNet	99.19	99.39	1.20	81.00	80.00

**Table 6:** Recognition results of different classification models.

Model Name	Accuracy ± Std	Precision ± Std	Recall ± Std	F1-Score ± Std	MCC ± Std	FPR ± Std	FNR ± Std	NPV ± Std	FDR ± Std	Specifity ± Std	AUC ± Std
CBAM-DenseNet	98.7104 ± 0.0556 ^b^	97.1897 ± 0.8751 ^b^	97.1251 ± 0.8817 ^b^	97.1267 ± 0.8794 ^b^	96.0207 ± 0.0371	0.8572 ± 0.0004 ^b^	3.1577 ± 0.0015 ^b^	99.1839 ± 0.0414 ^b^	3.0911 ± 0.0014 ^b^	99.1784 ± 0.0730 ^b^	0.9986 ± 0.0003 ^a^
DSA-DenseNet	99.1959 ± 0.1589 ^a^	98.2318 ± 0.8567 ^a^	98.2119 ± 0.8666 ^a^	98.2088 ± 0.8641 ^a^	97.4292 ± 0.1729 ^a^	0.5069 ± 0.0006 ^a^	2.1057 ± 0.0023 ^a^	99.4451 ± 0.1232 ^a^	2.0814 ± 0.0038 ^a^	99.5286 ± 0.1196 ^a^	0.9992 ± 0.0007 ^a^
ECA-DenseNet	98.9826 ± 0.1557 ^ab^	97.3193 ± 0.8773 ^ab^	97.2912 ± 0.8872 ^ab^	97.2899 ± 0.8823 ^ab^	96.7225 ± 0.1002 ^ab^	0.6665 ± 0.0008 ^ab^	2.5560 ± 0.0015 ^ab^	99.2956 ± 0.1183 ^ab^	2.5295 ± 0.0025 ^ab^	99.3430 ± 0.0907 ^ab^	0.9993 ± 0.0008 ^a^
ICBAM-DenseNet	98.7380 ± 0.3021 ^b^	96.8659 ± 1.4817 ^b^	96.8720 ± 1.4953 ^b^	96.8684 ± 1.4885 ^b^	95.9607 ± 0.2426 ^b^	0.8513 ± 0.0019 ^b^	3.1570 ± 0.0046 ^b^	99.0967 ± 0.1244 ^b^	3.1643 ± 0.0072 ^b^	99.1462 ± 0.2037 ^b^	0.9987 ± 0.0014 ^b^
SE-DenseNet	98.9746 ± 0.0657 ^ab^	96.9776 ± 0.5794 ^b^	96.9772 ± 0.5829 ^b^	96.9755 ± 0.5815 ^b^	96.7930 ± 0.0631 ^b^	0.6399 ± 0.0006 ^b^	2.5577 ± 0.0021 ^b^	99.2909 ± 0.0891 ^b^	2.5590 ± 0.0016 ^b^	99.3530 ± 0.0681 ^b^	0.9981 ± 0.0006 ^a^
CBAM-DBIRNet	98.6441 ± 0.1238 ^b^	96.6187 ± 0.5400 ^b^	96.6157 ± 0.5400 ^b^	96.6071 ± 0.5389 ^b^	95.7692 ± 0.0767 ^b^	0.9212 ± 0.0009 ^b^	3.3065 ± 0.0042 ^b^	99.1018 ± 0.0618 ^b^	3.3054 ± 0.0034 ^b^	99.0585 ± 0.0677 ^b^	0.9982 ± 0.0006 ^a^
ICBAM-DBIRNet	98.8375 ± 0.0980 ^b^	97.4549 ± 0.7489 ^b^	97.4475 ± 0.7506 ^b^	97.4471 ± 0.7498 ^b^	96.4768 ± 0.0847 ^b^	0.7936 ± 0.0007 ^b^	2.7066 ± 0.0022 ^b^	99.2475 ± 0.0702 ^b^	2.6994 ± 0.0019 ^b^	99.1447 ± 0.1349 ^b^	0.9983 ± 0.0007 ^a^
SE-DBIRNet	98.2360 ± 0.0859 ^c^	96.0783 ± 0.9970 ^c^	96.0709 ± 0.9970 ^c^	96.0548 ± 0.9951 ^c^	94.6371 ± 0.1090 ^c^	1.2865 ± 0.0009 ^c^	4.0614 ± 0.0054 ^c^	98.9300 ± 0.0780 ^c^	4.0518 ± 0.0042 ^c^	98.6974 ± 0.1161 ^c^	0.9981 ± 0.0009 ^a^

Note: Results are mean ± std over 10-fold cross-validation. Different superscript letters within a column indicate statistically significant differences (paired *t*-test with Bonferroni correction, α = 0.00179 for 28 comparisons). Models sharing the same letter are not significantly different. For AUC, no significant differences were found among any models (all share ‘a’).

**Table 7:** Performance comparison of the proposed model with state-of-the-art models.

Model Name	Accuracy ± Std	Precision ± Std	Recall ± Std	F1-Score ± Std	Total Parameter Count	GFLOPs	Model Size (MB)
DenseNet201	96.4316 ± 0.9573 ^b^	96.4773 ± 0.9500 ^a^	96.4316 ± 0.9573 ^a^	96.4339 ± 0.9549 ^a^	20,305,989	4.20	77.4600
EfficientNetB0	96.5671 ± 0.8103 ^b^	96.6138 ± 0.7914 ^a^	96.5671 ± 0.8103 ^a^	96.5681 ± 0.8104 ^a^	4,049,571	0.78	15.45
InceptionV3	94.7378 ± 1.3479 ^c^	94.7690 ± 1.3382 ^c^	94.7378 ± 1.3479 ^c^	94.7351 ± 1.3533 ^c^	21,802,821	5.73	83.18
MobileNetV2	95.1215 ± 1.2079 ^c^	95.1941 ± 1.1887 ^c^	95.1215 ± 1.2079 ^c^	95.1165 ± 1.2038 ^c^	2,265,893	0.30	8.64
ResNet50	96.5441 ± 0.9315 ^b^	96.5770 ± 0.9333 ^a^	96.5441 ± 0.9315 ^a^	96.5387 ± 0.9366 ^a^	23,591,813	4.09	90.02
VGG16	94.7375 ± 1.1979 ^c^	94.7843 ± 1.1772 ^c^	94.7375 ± 1.1979 ^c^	94.7328 ± 1.2043 ^c^	134,303,045	15.50	512.32
Xception	94.6701 ± 1.2062 ^c^	94.7061 ± 1.1699 ^c^	94.6701 ± 1.2062 ^c^	94.6683 ± 1.1976 ^c^	20,867,181	8.40	79.61
VGG19	94.5571 ± 0.9502 ^c^	94.6537 ± 0.9482 ^c^	94.5571 ± 0.9502 ^c^	94.5593 ± 0.9504 ^c^	143,667,269	19.60	548.11
SE-DBIRNet	98.2360 ± 0.0859 ^a^	96.0783 ± 0.9970 ^a^	96.0709 ± 0.9970 ^a^	96.0548 ± 0.9951 ^a^	2,847,381	0.89	10.86

Note: Different superscript letters within a column denote statistically significant differences (paired *t*-test with Bonferroni correction, α = 0.00139). Models with the same letter are not significantly different. For total parameter count, GFLOPs, and model size, these values are deterministic (architecture-specific) and therefore no statistical comparison is applicable.

**Table 8:** Performance evaluation of SE-DBIRNet on the independent dataset.

Fold	Accuracy (%)	Precision (%)	Recall (%)	F1-Score (%)	MCCA (%)	FPRA (%)	FNRA (%)	NPVA (%)	FDRA (%)	SpecificityA (%)	AUC
1	100.0000	100.0000	100.0000	100.0000	100.0000	0.0000	0.0000	100.0000	0.0000	100.0000	1.0000
2	99.8252	99.6552	99.6503	99.6503	99.5369	0.1155	0.3497	99.8829	0.3448	99.8845	1.0000
3	100.0000	100.0000	100.0000	100.0000	100.0000	0.0000	0.0000	100.0000	0.0000	100.0000	1.0000
4	100.0000	100.0000	100.0000	100.0000	100.0000	0.0000	0.0000	100.0000	0.0000	100.0000	1.0000
5	100.0000	100.0000	100.0000	100.0000	100.0000	0.0000	0.0000	100.0000	0.0000	100.0000	1.0000
6	100.0000	100.0000	100.0000	100.0000	100.0000	0.0000	0.0000	100.0000	0.0000	100.0000	1.0000
7	100.0000	100.0000	100.0000	100.0000	100.0000	0.0000	0.0000	100.0000	0.0000	100.0000	1.0000
8	100.0000	100.0000	100.0000	100.0000	100.0000	0.0000	0.0000	100.0000	0.0000	100.0000	1.0000
9	99.6504	99.3175	99.2982	99.2981	99.0734	0.2328	0.7018	99.7693	0.6825	99.7672	1.0000
10	99.6479	99.3175	99.2982	99.2982	99.0734	0.2328	0.7018	99.7650	0.6825	99.7672	0.9999

## Data Availability

The image dataset of mango varieties used in this study is publicly available on Mendeley Data (doi:10.17632/5MC3S86982.1). The dataset can be accessed and downloaded directly via the following link: URL https://data.mendeley.com/datasets/rk4mm635rn/1, DOI: 10.17632/rk4mm635rn.1, accessed on 15 June 2026. The dataset and code used to support the findings of this study are available in the Google Drive repository at: https://drive.google.com/drive/u/0/folders/1Hh1GzgzhXKGQjk5HZzsvSGFkfM0zIy_-, accessed on 15 June 2026.
